# A Review of Lightweight Self-Healing Concrete

**DOI:** 10.3390/ma15217572

**Published:** 2022-10-28

**Authors:** Feng Huang, Shuai Zhou

**Affiliations:** 1 College of Materials Science and Engineering, Chongqing University, Chongqing 400045, China; 2 State Key Laboratory of Mountain Bridge and Tunnel Engineering, Chongqing Jiaotong University, Chongqing 400074, China; 3 School of Civil Engineering, Chongqing Jiaotong University, Chongqing 400074, China

**Keywords:** composites, lightweight aggregate self-healing concrete, self-healing materials

## Abstract

Cementitious composites often crack because of their low tensile strength. The ability of self-healing cementitious composite to automatically repair cracks has attracted widespread attention. Lightweight aggregate (LWA) has a low density and a high porosity which can provide storage space for a healing agent. The healing mechanisms and healing compositions of lightweight self-healing concrete (LWSHC) have been summarized in this research. The workability, compressive strength, crack repairing, and durability of LWSHC performance is also illustrated. A LWA with interconnected pores and a high strength should be integrated into LWSHC to increase the crack closure rate and mechanical properties. Expanded perlite is the most suitable LWA carrier for bioremediation. The chemical healing agents are better than the biological healing agents at present since the biological healing agents have more negative effects. A sodium silicate solution is a good choice as a chemical healing agent. Vacuum conditions, high-temperature processing, and the use of coating technologies on LWAs can improve the healing effect of LWSHC. The addition of fibers also enhance the self-healing ability of LWSHC. Further, the use of numerical simulation supports the healing performance of LWSHC. The goal of this research is to investigate the most appropriate component of LWSHC to ensure a high crack closure rate, strength healing ratio, and great durability while being lightweight. It can then be adopted in high-rise and large-span concrete structures to extend the service life.

## 1. Introduction

Cementitious composites are currently widely used. However, due to the low tensile strength of concrete, cracks are inevitable. These cracks come from plastic shrinkage, drying shrinkage, chemical shrinkage, thermal stress, external load, the expansion of ettringite, and the coupling effect of multiple factors, causing significant durability and structural problems. It is difficult to heal the microcracks within concrete using traditional repairing methods. In addition, implementing the real-time monitoring of cracks also imposes a huge burden on construction costs. Self-healing technology has been introduced as a sustainable method for repairing cracks, extending the service life of structural concrete, and reducing maintenance costs. It does not require the real-time manual maintenance of concrete and can repair cracks autonomously [1]. Therefore, in the past few decades, the application of self-healing technology has received extensive attention from scholars [2,3,4].

Self-healing technology in a cementitious composite is generally divided into the following three categories: (1) The use of auxiliary cementitious materials for repairing by directly adding mineral admixtures, crystalline admixtures, and polymer materials into concrete. (2) The use of carriers with healing agents for self-healing [5] where the repairing agents are stored inside the capsule walls of micro- or macroscopic capsules. In the case of changes in pH and other damaging factors, the capsule wall is torn, and the inner healing agent is released to play a self-healing role. (3) Adding microorganisms and nutrients into concrete, for example, bacteria can produce calcium carbonate to repair concrete cracks.

Self-healing concrete needs pores to carry these healing agents. Lightweight aggregate concrete (LWC) has a high porosity, which can provide storage space for healing agents. A LWA releases the healing agent when the crack propagates to the pores and has the advantage of being lightweight. With a parametric design, it can be used in high-rise modern structures and large-span bridges as a building material [6]. The application of LWAs in self-healing concrete has good prospects. A well-designed LWSHC can extend the service life of those concrete structures while saving on maintenance costs and time. However, to the best of our knowledge, no related review has summarized the current technological developments of LWSHC to date.

This review summarizes the application of LWAs in the field of self-healing concrete, aiming to elevate the self-healing performance of LWSHC. The self-healing mechanisms and the healing components of LWSHC are analyzed in Section 2. In Section 3, the self-healing performance of LWSHC is concluded. Section 4 displays the theoretical prediction of the healing behavior of LWSHC. Finally, the conclusions of the present study are summarized in Section 5.

## 2. Components and Mechanisms of Lightweight Aggregate Self-Healing Concrete

### 2.1. Lightweight Aggregate

**Figure 1:**
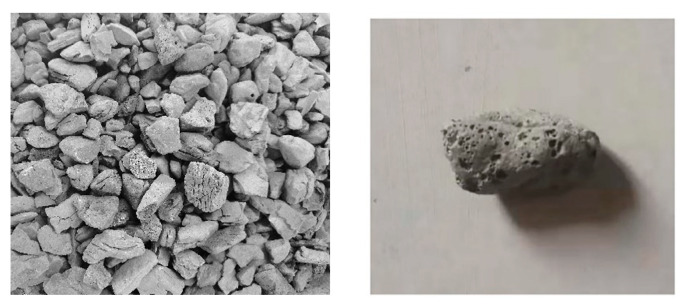
The morphology of LWAs.

**Figure 2:**
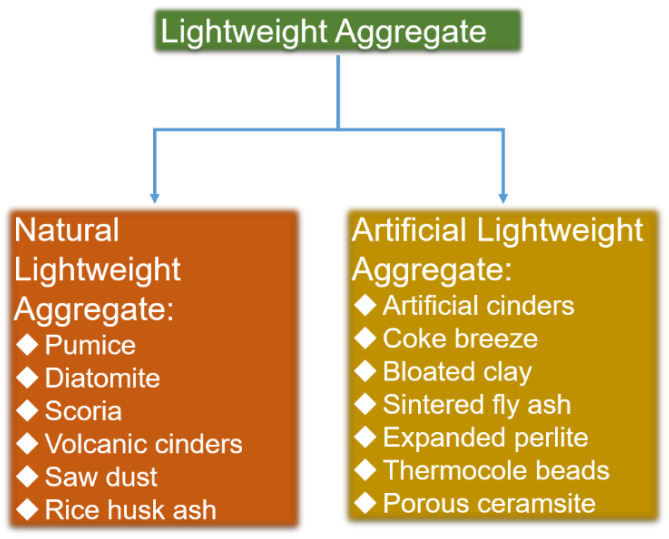
The classification of LWAs.

Previously, the main purpose of using LWC was to reduce the load of the concrete structure and the size of the load-bearing members [7]. Figure 1 displays the morphology of LWAs used in LWC. LWAs contain a lot of pores. Those pores provide space for storing the healing agents in LWSHC. LWA can generally be divided into natural aggregates and artificial aggregates [8]. Figure 2 lists the types of LWAs [9]. Although the cost per cubic meter of LWC may be greater than that of ordinary concrete, the overall cost of the structure with LWC is still less than that of ordinary concrete structures due to the reduced dead weight and lower foundation costs [10]. Many have scholars tested the properties of LWAs and found that the use of artificial aggregates in LWC had both economic and ecological benefits [11].

**Figure 3:**
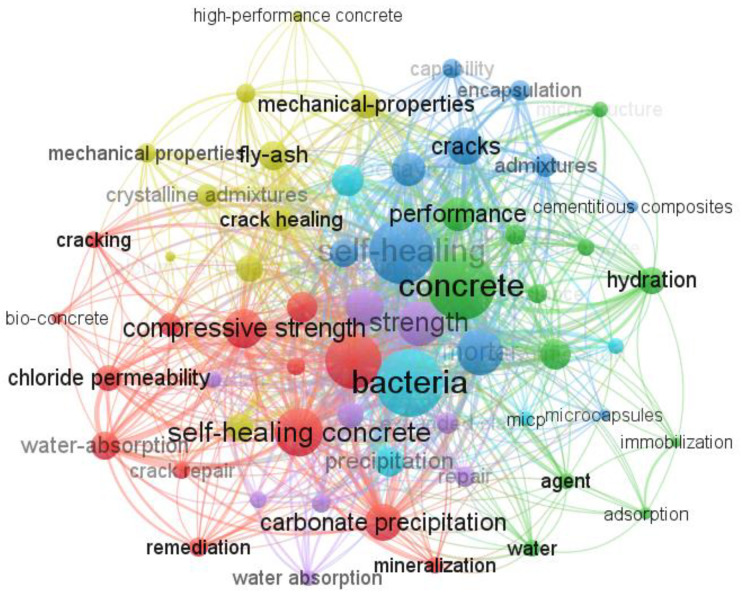
The network visualization of different keywords of LWSHC.

Meanwhile, a lot of studies focused on the development of LWSHC using LWAs. Figure 3 is the network visualization of different keywords of LWSHC. The bibliographic information was extracted from Scopus, one of the largest databases for academic abstracts and citations, using “lightweight aggregate self-healing concrete” as the keyword. The open-source tool Vosviewer was applied to draw Figure 3. “Bacteria” appeared 35 times and “expanded perlite” appeared 25 times. This means that the self-healing of LWSHC is mostly carried out by means of microbial self-healing. The most widely used LWA is expanded perlite.

It has been proven that using LWAs as carriers for healing agents in self-healing concrete is valid; damaged concrete was repaired with LWAs [12,13,14,15,16]. Stuckrath et al. [12] impregnated LWAs with chemical and biological solutions as healing agents in concrete mixtures. Then, a sulfoaluminate-based expansion agent, crystalline admixtures, and calcium hydrogen phosphate as a part of the cementitious material were adopted as the healing components, and the porous ceramsite was applied as the carrier of the sodium carbonate solution to study the self-healing potential of LWSHC [13,14]. Huynh et al. [15] adopted LWAs as protective carriers for bacillus subtilis natto to evaluate the self-healing ability of LWSHC by its biomineralization. Yu et al. [16] used rubber crumbs and hollow fly ash hollow microsphere particles as the LWA to reduce the density, matrix toughness, and crack width to produce lightweight, high-strength engineering cementitious composites with enhanced self-healing capabilities. A large number of scholars have studied the self-healing of LWSHC by impregnating healing agents. Table 1 lists the healing agents and LWAs used in LWSHC.

### 2.2. Chemical Healing Agents + LWA

The self-healing of cementitious materials can be divided into autogenous healing and autonomous healing [45]. Autogenous healing means that the self-healing of the mixture is achieved in the absence of other ingredients. Meanwhile, autonomous healing depends on a healing agent other than the cementitious composition. In both types of self-healing, self-healing is observed to significantly reduce crack width and water permeability. Chemical healing agents (e.g., sodium silicate and sodium carbonate) can enhance self-healing. The main cause of autogenous healing is attributed to multiple factors, such as hydration of unhydrated particles, matrix swelling, plugging of cracks by impurities and particles, or the precipitation of calcium carbonate. Hydration products were observed to repair large cracks, and the maximum repairable crack width was limited to 50–150 μm [46]. In addition to its hydration, the precipitation of calcium carbonate is believed to be capable of autogenous crack healing. The precipitation of calcium carbonate is mainly due to the reaction between calcium ions from matrix emissions and carbonates from carbon dioxide dissolution. The calcium carbonate precipitation process is the hydration of the cement paste, the precipitation of calcium carbonate crystals, and the blockage of the flow path due to the deposition or the movement of detached concrete fragments [47]. However, the bond between the concrete and CaCO_3_ formed by this process is unstable [48]. In the presence of water, the bonding performance of CaCO_3_ will be better. CaCO_3_ can fill 0.1 mm-wide cracks, which also indicates that the self-healing system of cement-based materials may be influenced by environmental factors.

**Figure 4:**
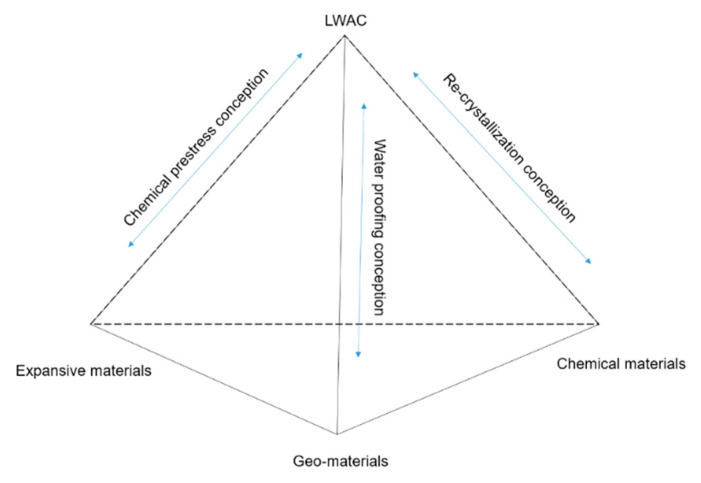
Schematic diagram of the self-healing mechanism of various mineral admixtures [49].

The self-healing properties of cement-based materials are greatly affected by their healing agents. Chemical repairing agents such as calcium carbonate, sodium silicate, CaHPO_4_·2H_2_O, calcium sulfoaluminate-based expansion agents, crystalline additives, and auxiliary cementing materials, have significantly improved performance as admixtures when they partially replace cement components [49,50]. They can provide silicon or aluminum for cement hydration. Figure 4 is a schematic diagram of the self-healing mechanism of each mineral admixture.

#### 2.2.1. Water as the Healing Agent

Water is one of the indispensable factors affecting self-healing effects [51]. During the hydration of cement, both calcium hydroxide and carbon dioxide dissolution depend on the presence of water. However, due to the lack of free water supply within hardened concrete, most of the hydration products, such as calcium hydroxide and calcium carbonate, can only form on the surface of the concrete and are thus susceptible to leaching [52]. Therefore, to ensure the healing of internal cracks, a “pool” of free water should be provided in the concrete. Additional water, such as that provided by LWAs, can increase the degree of hydration, resulting in the formation of more C-S-H gels. Only a little cement remains unhydrated, while monosulfate tends to form a loose structure, resulting in microcracks after loading [53]. The addition of zeolite particles can effectively elevate the self-healing effect of cement-based composites as the internal curing agent releases additional water for secondary hydration [18]. Furthermore, the compressive strength of the cement-based composite with 30% zeolite was increased by 15%. Controlling a certain water absorption rate of zeolite can maximize the self-healing rate. Too much water will change the water-cement ratio and affect the fluidity and strength of concrete. The pre-wetting treatment of LWAs can only improve the self-healing properties of concrete to some extent. As a source of moisture in an environment lacking moisture, the pre-wetting treatment of LWAs can not only promote the reaction by providing water for hydration but also provide a good nutrient reservoir for the cells within the LWA [54]; the microorganisms can be provided with sufficient water for mineralization, which raises the self-healing properties of LWSHC. Bundur et al. [54] explored the use of pre-wetted lightweight expanded shale aggregates as an internal nutrient reservoir for microorganisms in LWSHC. Compared with neat mortars containing pre-wetted LWAs, self-healing bacterial mortars containing pre-moistened LWAs had higher strength.

#### 2.2.2. Sodium Silicate as the Healing Agent

**Figure 5:**
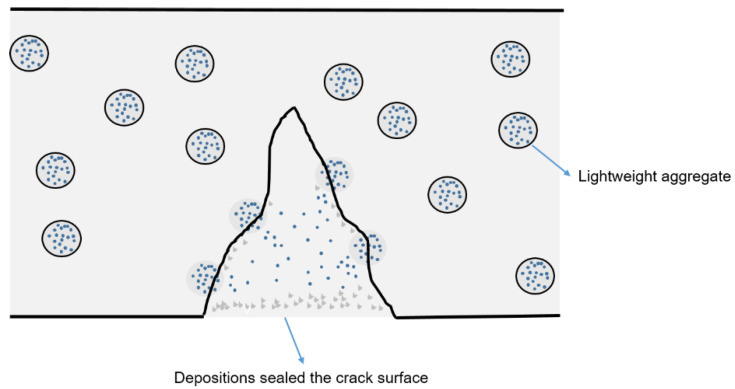
Schematic diagram of self-healing of sodium silicate-impregnated LWC [20].

The technology of impregnating LWC has been widely used [55,56]. Sodium silicate (Na_2_SiO_3_) can effectively increase the elastic modulus of concrete when encapsulated with different microcapsules [30,31,57]. The schematic diagram of LWSHC with a sodium silicate-impregnated LWA is displayed in Figure 5.

It is well known that C-S-H gel, as the main reaction product of Portland cement hydration, determines most of the properties of cement. Once sodium silicate is released from the impregnated LWA, it will react with calcium hydroxide (a product of cement hydration) to form a C-S-H gel, thereby restoring strength. The relevant chemical reaction is as follows [58]:

(1) $${\mathrm{Na}}_{2}{\mathrm{SiO}}_{3}+\mathrm{Ca}{\left(\mathrm{OH}\right)}_{2}\to x\left(\mathrm{CaO}\cdot {\mathrm{SiO}}_{2}\right)\cdot H_{2}O+{\mathrm{Na}}_{2}O$$

Sodium silicate as a self-healing agent has a significant effect on restoring strength and improving durability. By using a sodium silicate solution, the impregnation of LWAs can create a self-healing effect in LWSHC. The impregnation of LWA particles with sodium silicate raised the strength recovery by more than five times and reduced capillary water absorption by nearly half [20].

There are many new methods for improving the impregnation effect. The absorption rate was significantly improved under vacuum conditions compared with immersion under atmospheric conditions [59]. Coarse LWAs with high porosity were usually adopted. The absorption rate of LWAs in a sodium silicate solution increased to 31% after vacuum immersion for 30 min in its immersion process while the absorption rate of LWAs after immersion for 3 days reached 19%. In order to prevent excess sodium silicate from leaking from the aggregate or interacting with the cement matrix prematurely, the impregnated LWA was coated with polyvinyl alcohol (PVA) by spraying [20]. Thus, the reduction in subsequent healing performance can be effectively avoided. New types of LWAs may contribute to the healing effect. Rashid et al. [21] developed a new kind of lightweight fly ash foam. They made lightweight ceramic microbeads wrapped with a sodium silicate solution for LWSHC. With the ceramic microbeads, the self-healing ability rose greatly.

#### 2.2.3. Sodium Carbonate as the Healing Agent

The addition of carbonates to clay-like LWAs increased the content of calcium ions, and the strong chemical gradient between cement and clay promoted the formation of C-S-H gels and other white precipitation products [29,60]. In this way, self-healing is initiated.

Wang et al. [19] used liquid sodium carbonate to impregnate lightweight clay aggregates for the self-healing of cement-based composites. The results showed that, although the penetration of sodium carbonate significantly reduced the anti-polarization ability of the concrete, the self-healing performance and hardening properties were improved. The 28-day-compressive strength of self-healing samples was about 7.5% greater than the value of control samples. SEM observations proved that the healing products existed in the interior and the crack surface. The crystals obtained on the surface were mainly composed of calcite.

When sodium carbonate is retained as a healing material in the interior of the LWA, it is necessary to avoid excessive consumption of sodium carbonate by early hydration. During the stirring and curing process, there will be a certain amount of exudation, which contained about 50% sodium carbonate after 7 days [61]. Yang et al. [50] adopted the technology of encapsulating LWAs with a built-in sodium carbonate solution and using an epoxy curing agent. The self-healing trigger rate of cracks in LWAs under this technology reached 91.23%. Although at high temperatures, it can still effectively protect the healing agent. Hence, sodium carbonate repaired the cracks. The encapsulation technology of a sodium carbonate solution in LWAs has an important influence on its self-healing performance. 

Wang et al. [13] compared LWAs with mineral additives and built-in carbonates. During encapsulation, the epoxy coating interacted with them after being embedded in the cement, withstanding the complex stress of concrete. The mechanical properties and the preservation ability of carbonate were promoted. The cracks had a relatively obvious filling effect after the occurrence of cracks which manifested as a better self-healing ability. The self-healing test showed that the self-healing speed became faster for the treated specimen. The self-healing of cracks occurred both internally and on the surface in previous studies [13]. Hence, the coating technology is a good choice for chemical healing agents in LWSHC.

### 2.3. Biological Healing Agents + LWA

**Figure 6:**
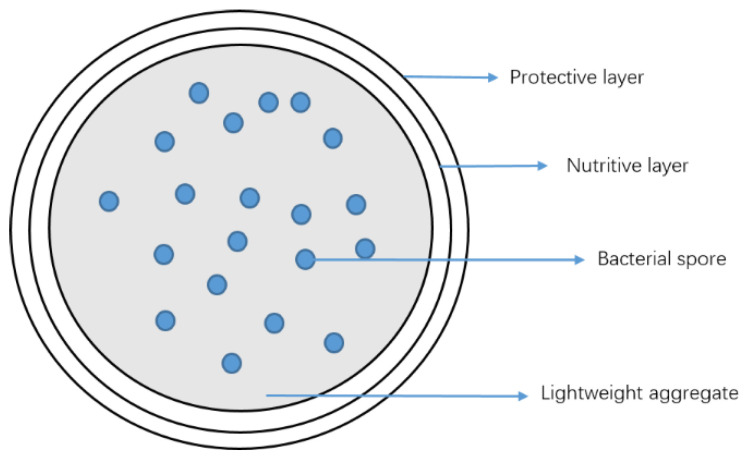
Schematic diagram of a LWA as a microbial carrier [62].

The research on self-healing concrete is often carried out by encapsulating bacteria in carriers. The most commonly used carriers are a polymer material, LWA, cementitious material, special minerals, nanomaterial, and waste-derived biomass [62]. The carrying performance of LWAs for bacteria is discussed here. The pores of LWAs can be well used as a carrier for microorganisms to achieve self-healing. Figure 6 is a schematic diagram of a LWA as a microbial carrier.

Different kinds of biological healing agents have different detailed healing mechanisms. A large number of recent studies have focused on the self-healing of microorganisms by using microorganisms to induce biomineralization and protection of microorganisms with LWAs. Biomineralization involves a series of biochemical reactions in which microorganisms initiate mineral precipitation. Microorganism-induced calcium carbonate precipitation (MICP) is an example of the biomineralization process, which is widely used in self-healing concrete [63,64,65,66,67,68,69,70,71]. The goal of MICP is to automatically repair cracks in the cement-based matrix, and the sediment from MICP can be used to bind particles to form composites or seal cracks in concrete. Research on biomineralization in concrete has shown the possibility of self-healing, such as the sealing of cracks, recovery of toughness, and compressive strength. It has been proven that biomineralization can significantly reduce permeability by filling cracks in concrete. One of the main challenges for the application of biomineralization in cement-based materials is the restrictive environment, such as a high pH, lack of moisture, and low nutrient concentrations. Since the microorganisms need to meet the harsh living environment, such as the high alkalinity of concrete, the implementation cost of bacterial concrete is quite high, mainly due to the cultivation, transportation, and protection of the living cells and their respective nutrients. Therefore, further requirements will need to be made for the system [52]. A low pH of the cementitious matrix was sought. The healing of LWSHC peaked when the microorganisms were protected with low-alkaline cement (i.e., potassium magnesium phosphate cement) on the expanded perlite particles encasing bacterial spores. Meanwhile, the water applied in the cementitious matrix is also under some requirements. Using a nutrient solution instead of concrete mixing water is a method to introduce the required nutrients [68]. However, the water in the hydration process will decrease, which requires the water-cement ratio to be 0.45 or above [69]. Otherwise, it may lead to cell death. Further research is needed to determine the most appropriate method of incorporating bacteria into cracked concrete. LWAs have been selected to protect them; different LWAs and different microorganisms in LWSHC have been investigated by scholars.

#### 2.3.1. Different Lightweight Aggregates

The advantages and limitations of various carriers were discussed in previous research [72]. The bacterial carrier should meet two general requirements: (1) its strength should be sufficient to withstand the mixing and hardening of concrete and (2) its mechanical properties should be comparable to those of the concrete matrix to ensure that cracks activate biological substances. The self-healing mechanisms of different microbial carriers are different for the various carriers used [73]. Wang et al. [74] proposed a biohydrogel to protect microorganisms and provide moisture. They found that the self-healing performance of the mortar containing the biohydrogel was better than that of the mortar containing the pure hydrogel without bacteria. Meanwhile, Pungrasmi et al. [75] used sodium alginate hydrogel microcapsules in their experiments. They compared the viability of bacteria encapsulated by several techniques (extrusion, spray drying and freeze drying), and found that the frozen packaging effect was the best. Zamani et al. [76] applied a polyurea polymer as a carrier for Pseudomonas sclerotiorum. However, given the additional cost and complicated process required to produce bacterial carriers, the above-mentioned methods to enhance microbial viability have limitations. As an alternative method to protect bacteria in concrete, LWAs with a lower cost, easier treatment, and wider application have also been explored by many scholars as bacterial carriers in LWSHC.

LWAs prepared from clay and other raw materials were widely adopted as bacterial carriers [25,77,78,79]. Zhang et al. [78,79] demonstrated the feasibility of expanded perlite as a novel bacterial carrier to quantify crack healing by immobilizing Bacillus gaurnii. Comparisons were made with two other immobilizations, namely, the direct introduction of bacteria and expanded clay immobilization of bacteria. The results indicated that samples of expanded perlite-immobilized bacteria exhibited the most effective crack healing. Compared to previous research on perlite, vermiculite, zeolite, and silica supports [80], the results showed that expanded perlite was the most suitable carrier for bioremediation. The LWAs by Chen et al. were made by crushing and firing natural shale at 1100–1200 °C [41]. The surface was porous and irregular, with a particle size of 1–8 mm, a specific gravity of 0.99 g/cm^3^, and a porosity of 24.97%. The use of a LWA as a carrier can effectively protect Pasteurella to induce calcium carbonate precipitation. In addition, diatomaceous earth is resistant to high pH environments and can protect bacteria [81]. Bundur et al. [54] studied the internal nutrient reservoirs to enhance cell viability in mortars by pre-wetting lightweight finely expanded shale aggregates. The addition of the internal nutrient pool resulted in an increase in the remaining vegetative cells without a substantial loss of strength. Lightweight expanded shale aggregates were also used as fine aggregates. Studies indicated that, especially in low water-to-binder ratio concrete, the introduction of an internal nutrient reservoir was an effective method to increase the appearance of nutrient cells. In conclusion, LWAs can effectively solve the above two generally satisfied requirements and maintain good properties. In addition, some special treatments on LWAs are good for improving their properties, i.e., after being treated at high temperatures [82], porous ceramsite achieved good properties. LWAs can increase porosity and enhance the healing ability [25], while artificially processed LWAs such as modified ceramsite can effectively avoid the influence of cement [43]. Furthermore, modified LWAs reduce water absorption and provide better mechanical support and interface properties of LWSHC.

#### 2.3.2. Different Microorganisms

Table 2 shows that different microorganisms in LWAs have different properties, mainly in the number of generated cells. They affect the content of mineralized substances, which puts forward requirements for the bearing capacity and porosity of LWAs. Salehi et al. [35] compared the activity of four microbial strains (i.e., Sporosarcina Pasteurii, Bacillus Megateterium, Sporosarcina Ureae, and Bacillus Licheniformis) at a cell concentration of 10^7^ cells per milliliter on calcium carbonate precipitation. Three different fiber percentages (0%, 0.5%, and 1%) were adopted. Experimental results showed that adding bacteria and fibers simultaneously was effective and improved the compressive strength, tensile strength, and flexural strength. It also reduced the water absorption and permeability of the concrete in the sample. A comparison of bacterial samples showed that Bacillus Licheniformis and Bacillus Megateterium were superior to Sporosarcina Pasteurii and Sporosarcina Ureae in inducing calcium carbonate precipitation in LWSHC. In addition, microscopic analysis was carried out on the formed products. Calcium carbonate was formed in the cracks. The crystalline phase of calcite was characterized, indicating that calcium carbonate often existed in the form of calcite and vaterite. The results agreed with the study of different microbial self-healing technologies by Wasim et al. [83]. The nutrients and environment in favor of the growth of microorganisms can improve the degree of microbial response, but sufficient microorganisms are still needed to heal the cracks. Alazhari et al. [64] studied the number of bacterial spores, using expanded perlite to immobilize bacterial spores. Healing was achieved when the expanded perlite replaced up to 20% of the fine aggregate in concrete and a suitable ratio of spores to calcium acetate was provided. Research has proven that self-healing requires not only sufficient healing compounds but also a proper number of bacterial spores to ensure that enough cells are involved in the self-healing process. Based on Table 2, Bacillus alcalophilus has the highest cell concentration among different types of microorganisms in LWSHC [43].

## 3. Performance of Lightweight Aggregate Self-Healing Concrete

**Figure 7:**
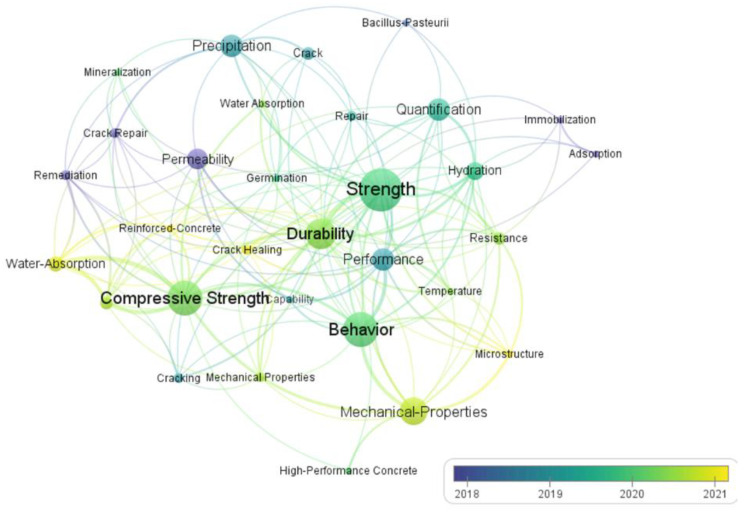
The network visualization among different keywords of self-healing performance.

In the preparation of LWSHC, the addition of minerals and treated LWAs influences the performance of the concrete. Figure 7 is a network visualization among different keywords of self-healing performance in the literature. The bibliographic information was extracted from Scopus, using “lightweight aggregate self-healing concrete AND performance” as the keyword. The open-source tool Vosviewer was adopted to draw Figure 7. There are many studies on the impact of self-healing performance. In terms of study, research on the water absorption, mechanical properties, and self-healing efficiency of LWAs is popular. The keywords of strength, durability, and self-healing performance appear more frequently, indicating that scholars pay more attention to those aspects. On this basis, this section discusses the workability, crack closure ability, mechanical properties, and durability of LWSHC.

### 3.1. Workability

The workability of fresh concrete decreased due to the aggregate absorbing a large amount of mixing water [84]. The mean water absorption for tested composites was up to 16.7% for reference concretes with non-impregnated LWAs [84]. Therefore, the workability of LWC also needs to be paid attention to when using impregnation.

When an external healing agent is applied, it may lead to changes in the properties of concrete, such as a change in workability. This change affects cement hydration, pore size, and porosity [85]. Kim et al. [86] investigated the biological calcification metabolism of vegetative cells and the influence of nutrients on hydration kinetics. Hydration was affected by nutrients. The dissolution of the cement was inhibited and thus the hydration was delayed. Therefore, when the nutrients in the LWA flowed out, it was more likely to inhibit the hydration of the cement, increasing workability. This puts forward requirements for the packaging technology when LWAs are adopted as carriers. Workability also needs to be considered when using calcium carbonate and sodium silicate for dipping. In the study, the fluidity test of the impregnated LWC mixed with mineral admixtures and carbonates showed that the fluidity of experimental groups with added substances was 184.33–212.50 mm, which was greater than the 179.03 mm of the control group [19]. This indicates that the fluidity of the mixture increased after the addition of the self-healing admixture system. Although the sodium carbonate solution continued to seep out of the LWA, the amount of solution seeping out from the three samples was almost the same. Therefore, the effects on workability were the same. The increase in fluidity was attributed to the presence of the Na_2_CO_3_ solution, which was consistent with the previous study [87]. As a surfactant, sodium carbonate dissolves in water to form a large amount of CO_3_^2−^, Na^+^, and OH^−^. OH^−^ first destroys the surface of some particles of the cementitious material and generates a large amount of Ca^2+^. CO_3_^2−^ and Ca^2+^ then generate calcium carbonate. It coats the surface of the unhydrated cementitious material, which raises the fluidity of the slurry. Therefore, in the case of LWAs and other substances loaded or wetted, the fluidity of concrete will not be reduced due to its higher porosity and more mixing water absorption. Increased fluidity may be caused by the outflow of solutions such as sodium carbonate that increase particle surface activity or inhibit hydration. Hence, the coating is needed when using LWAs and impregnation to avoid the influence on the workability of LWSHC. Upon crack formation, the coating is broken. The inner healing agents are released from the LWA particles and produce healing products, which then plug the cracks [20]. In order to prevent any potential leakage of the healing agents out of the aggregates or any premature interaction with the cementitious matrix, the impregnated aggregates can be coated using a spray gun to apply the coating. During the rotation of a disc pelletizer, the aggregates were sprayed with the coating solution with simultaneous drying by blowing a stream of hot air. Thereafter, the encapsulated LWAs impregnated with healing agents were stored in an airtight plastic container until used in the concrete mixes [20].

Different types of LWAs have different water absorption, which influences workability too. The water absorption of a LWA is affected not only by the physical and chemical properties of the material but also by the internal pore characteristics of the material to a certain extent. The expanded perlite and gravelly ceramsites have rough surfaces and micropores. Through the microscopic morphology analysis previously studied [8], it was found that there are many holes on the surface of gravelly ceramsite. However, its internal structure is compact and the internal pores are mostly closed. Hence, its water absorption capacity is limited. However, the internal structure of expanded perlite is loose and porous, and the pores are interconnected with each other, which makes it easier to store the water absorbed.

### 3.2. Crack Closure

With the development of self-healing technology, the width of healed cracks has risen. Different healing compositions of LWSHC have different crack closure rates. The crack closure rate is calculated by the reduced crack size after healing compared with the crack size before healing [13,17,20]. Table 3 exhibits the crack closure performance of LWSHC with different healing agents and LWAs. In the study by Wiktor and Jonkers [37], a two-component biochemical agent consisting of bacterial spores and calcium lactate was released from the particles through the crack. Expanded clay particles were adopted. Subsequent bacterial-mediated calcium carbonate formation resulted in the physical closure of the microcracks. The results showed that, after immersion in water for 100 days, the width of the healed cracks in the bacterial concrete reached 0.46 mm, while that in the control specimens was 0.18 mm. The observed doubling of healing potential was due to bacterial metabolic activity and was supported by oxygen profiling measurements. The result showed that O_2_ consumption came from the bacteria. Hence, the two-component healing agents can repair cracks effectively. Zhan et al. [17] used expanded vermiculite to immobilize microorganisms which can effectively repair cracks with a width of 400 µm after curing for 28 days. The expanded vermiculite had a layered structure, and there were a lot of gaps between the layers, which provided space for microorganisms. Therefore, the expanded vermiculites were an excellent carrier for immobilizing microorganisms. In reality, only some healing agents are released, while the rest remain in the LWA. The protection of LWAs is very important for healing agents. Through the combination of alkaliphilic bacteria of the genus Bacillus and expanded clay aggregates, the 0.4 mm-wide cracks were partially recovered after the load was removed [26]. After 28 days, the specimen cracks were closed, ranging from 10% to 90%, depending on the initial size of the crack in the concrete matrix. From Table 3, the 850 µm-wide cracks could be healed in LWSHC [36]. If the cracks are too large, they cannot be repaired by self-healing. The crack closure rate is significantly affected by the crack width and depth. The maximal crack closure rate of LWSHC can be up to 98.9% [17]. From the literature, the biological healing agents were more efficient than the chemical healing agents based on the crack closure rate and the healed crack width. However, the healing effect of the chemical agents was better than that of biological agents in previous research [12]. This contradiction might be attributed to the instability of the biological healing agents without coating. Since self-healing can repair cracks in the early stage [88,89], excessive cracks are avoided. Mineralization by encapsulating microorganisms can also achieve healing in concrete [90]. The healing agent showed an obvious color difference, such as bacterial mineralization producing a white precipitate [44]. This is consistent with the phenomenon of the white substance filling cracks visually observed in the literature [91,92]. However, there are limitations considering that internally healed cracks cannot be confirmed by visual monitoring methods, which are only capable of observing the surface of the specimen. There are also some studies showing that non-destructive testing using acoustic emission, ultrasound, and electromechanical impedance techniques [93,94] can identify damage in the structure and quantitatively evaluate the crack closure rate [95]. This is a development direction for the quantitative description of self-healing behavior. The healing performance of expanded clay particles with and without coating protection was different [32]. In the case of styrene-acrylic emulsion as a protective coating, the crack closure rate of uncoated expanded clay particles was 70%, and the crack closure rate of coated expanded clay particles was 75%. As a comparison, the crack closure rate of ordinary mortar was 50%. Self-healing with coated LWAs is more efficient. LWAs can be wrapped by different methods, such as silicone hydrophobic agents, aqueous epoxy resin, composite paste, and polyvinyl alcohol [17,20]. In terms of water absorption rate, the best treatment method was wrapped by composite paste [17].

Different types of LWAs have different inner structures which influence the crack closure rate of LWSHC. The interior of the expanded perlite is loose and porous and pores are interconnected [8]. The inside structure of the expanded vermiculite particles is layered, and there are gaps between folds [17]. Hence, more healing agents are stored in the LWA and the crack closure rate of LWSHC with those LWAs is high. On the contrary, the inner structure of gravelly ceramsite is dense [8]. Although there are pores in it, most of them are closed pores that are not connected. The healing agent is insufficient; hence, the crack closure rate is low. The LWSHC needs a LWA with interconnected pores to increase the crack closure rate.

### 3.3. Mechanical Properties

Many researchers rely on mechanical properties to verify the efficiency of self-healing (see Table 4, Table 5 and Table 6). Table 4 is a summary of the literature related to the compressive properties of LWSHC. The flexural properties of LWSHC are displayed in Table 5, while Table 6 shows the tensile properties. For LWSHC, the continuous hydration of calcium carbonate, sodium silicate, microorganisms, or itself can fill the microcracks, which decreases the stress concentration. Hence, the mechanical properties were improved. The incorporation of healing agents may affect its early strength. For example, the compressive strength was reduced by 50% after packaging microorganisms and calcium lactate yeast extract [79]. However, the strength recovered after damage due to self-healing. From Table 4, Table 5 and Table 6, it can be found that the strength is significantly improved after self-healing, but is also related to different repairing agents and LWAs. Balam et al. [33] used Sporosarcina pasteurii and LWAs of Leca to achieve self-healing. The system can effectively fill cracks. After adding active spore powder to the mortar, its 28-day compressive strength was increased by 34%, higher than that of normal mortar. When using the treated LWC with mixing water containing bacteria, it showed a higher compressive strength with an increase of 38% [33]. The fiber-contained LWC was subjected to water curing for 14d, 28d, 42d, and 56d after burning. Its average compressive strength, average elastic modulus, average splitting strength, and average fracture modulus were increased by 38.46%, 44.47%, 85.12%, and 25.21%, respectively [97]. The addition of fibers also improved the strength of concrete. The self-healing caused by water is related to the hydration process. Generally, the concrete is still hydrated within a few years, so its strength recovery also takes a long time. With water and LWAs, sufficient moisture ensured the progress of hydration, and the early self-shrinkage of LWC increased with the rise of its pre-wetting degree due to the moisture provided by pre-wetting [98]. Sani et al. [55] showed the role of sodium silicate in producing C-S-H gels. Concrete containing sodium silicate-impregnated LWAs and control samples were pre-cracked to a crack width of 300 µm. The strength recovery of the LWC impregnated with sodium silicate reached 80%, which was more than five times the recovery rate of the control specimen. At the same time, the compressive strength of the LWC-impregnated sodium carbonate also increased. The carbonate diffused from the carrier reacts with calcium ions in the pore solution, improving the compactness of the matrix; the calcium carbonate may also react with the aluminum phase to generate carboaluminate and hypocarbonate. The moisture was converted into crystal hydrate, which strengthened the connection between the hardened concrete and the calcium carbonate particles and raised the strength of repaired specimens [22,99]. The recovery of strength in the same LWC with different microorganisms was also different [35]. The compressive strength of LWSHC containing Bacillus Licheniformis, Bacillus Megateterium, Sporosarcina Pasteurii, and Sporosarcina urea was elevated by 25.61%, 20.13%, 16.80%, and 13.98%, respectively. The use of different LWAs also affects the mechanical properties [22]. Among the tested sterile culture protection methods, only zeolite and air entrainment resulted in a decrease in the compressive strength of the mortar. The remaining protection methods using diatomite, metakaolin, expanded clay, and granular activated carbon either increased or did not affect the original compressive strength. Kim et al. [86] found that microbial nutrients had a negative effect on the mechanical properties of concrete. It was deduced that the bacterial culture solution could negatively influence the dissolution of C_3_S or the production of hydration products. Organic substances could inhibit hydration, as they interfered with the contact of water and cement by combining with mineral particles. Further, the incorporation of ureolytic bacteria and nutrients in the mixing process could influence the change of phases and the ratio of hydration products, due to the metabolic activities of the ureolytic bacteria and the chemical degradation of nutrients during the hydration and hardening period. This will significantly lower the strength on all tested days, due to the low hydration rate from incorporating a large dosage of nutrients [86]. The biological self-healing system may have more negative impacts than the chemical self-healing system. However, Table 4, Table 5 and Table 6 indicate that a high content of the self-healing admixtures did not result in a significant reduction in flexural strength, tensile strength, and compressive strength [17,18,19]. Meanwhile, a slight improvement in strength development was likely due to the deposition of self-healing products from healing agents, therefore filling mortar pores and enhancing both compressive strength and split tensile strength [24]. Similarly, an increase in CaCO_3_ content and compressive strength occurred within the cement-paste samples when vegetative S. pasteurii cells were inoculated into cement paste with their nutrient medium. Thus, the strength increase in the bacterial mortars is attributed to the biomineralization phenomena overcoming the inherent strength reduction caused by replacing river sand with weaker LWAs [54]. Such opposite views may be caused by the unstable performance of biological healing agents. If the healing agent is effective, the negative impact will be suppressed and the strength of the LWSHC will increase. If the healing effect is not obvious, the strength will decrease due to the negative aspects of biological healing agents.

The strength healing rate is calculated according to η = σ_2_/σ_1_, where σ_1_ is the maximum stress for the virgin specimen and σ_2_ is the maximum stress for the healed specimen [20]. Considering the healing rate and stability, chemical healing agents are better than biological healing agents at present. From Table 4, Table 5 and Table 6, sodium silicate showed the highest healing rate of 80% [20]. The coated LWAs can increase the healing rate of LWSHC. The combination of diaphorobacter nitroreducens and expanded clay had the highest 28-day compressive strength of 67.8 MPa [22].

The failure of concrete is due to the propagation of cracks. In ordinary concrete with high-strength stone as the aggregate, the bearing capacity of the stone itself is high; the concentrated stress generated by crack propagation is not enough to damage the aggregate and the cracks often develop along the direction of the interface area. The strength of concrete mainly depends on the interface strength between the cement paste and aggregate. However, the strength of LWAs is lower than the strength of cement paste, which is the weak part. In this case, cracks will continue to develop through the LWA, and the LWA will be damaged. Therefore, the strength of LWSHC mainly depends on the strength of the LWA itself. The strength of LWSHC is affected by the aggregate strength; generally, the higher the aggregate strength is, the higher the corresponding concrete strength is. Clinoptilolite zeolite particles and expanded vermiculite have a high strength, which can increase the mechanical properties of LWSHC from Table 4, Table 5 and Table 6. The performance of LWAs is also very important and should have high strength characteristics.

### 3.4. Durability

LWSHC can have great durability due to its self-healing effect. With the hydration of cement particles and the evaporation of surface water, the humidity inside the concrete decreases continuously. When the relative humidity inside the cement paste is lower than that of the LWA, the water in the LWA migrates outward, which maintains a high relative humidity inside the cement paste for a certain time. The cement material inside the LWC is fully hydrated, and the aggregate plays an internal curing role, providing hydration water for cement particles during the curing period. Meanwhile, the microcracks are self-healed by healing agents, and a compact matrix enhances the durability of LWSHC. Compared with continuous water immersion, the LWSHC containing microorganisms has a better crack-sealing performance. When using bacillus subtilis and diatomite pellets [39], the water permeability of LWSHC decreased by 10–49%. When using Bacillus mucilaginous and ceramsite [44], the water permeability of the cracked LWSHC was reduced by 91–99%. When using alkaliphilic bacteria and expanded clay particles, increased crack sealing properties prevented the durability issues associated with microcracks [96]. In this way, the self-healing of the concrete was enhanced and the firmness was restored by 69–91%. After adding a healing agent to the mortar matrix, the recovery of compaction after cracking and exposure to water immersion and dry-wet cycles was assessed by water permeability tests. When subjected to wetting and drying cycles, the recovery of the compactness of the specimens with the healing agent increased significantly compared to the specimens without the healing agent [35]. By using Lysinibacillus boronitolerans and expanded clay, the water permeability of LWSHC was reduced by 70–75% [32]. By using Sporosarcina Pasteurii, Bacillus Megateterium, Sporosarcina Ureae, Bacillus Licheniformis, Leca coarse LWA, and Leca fine LWA, the water impermeability of LWSHC was recovered by 13.94–21.85% [35]. However, another study stated that the dry-wet cycle reduced the healing rate with biological healing agents [23]. This may occur since the biological healing agents are not stable. Due to the progress of the hydration reaction, the moisture inside the LWA decreases. The compaction of the concrete leads to the reduction in air. Meanwhile, the lack of oxygen and moisture required by the microorganisms slows down the self-healing effect. When using Sporosarcina pasteurii and expanded clay particles, the microbial treatment of LWAs can increase the resistance of concrete to chloride ion penetration by about 35% [33]. When using a Na_2_CO_3_ solution and lightweight clay aggregate, the self-healing admixtures system had a 16–20% reduction compared with that of the control group, considering the chloride diffusion coefficient [19]. The calcite deposition in the matrix of LWAC causes the structure of LWAC samples to be denser and the voids to be smaller [33]; hence, the chloride resistance is enhanced. Similar results were also obtained in other research when using a Na_2_CO_3_ solution and porous ceramsite [13]. The chloride ion migration coefficient of LWSHC was 21% less than that of LWC, suggesting that a healing effect occurs [13]. However, it caused a decrease in durability with a urea-CaCl_2_ solution. With increasing chloride ions, the depth of water penetration using bacteria in the mixed water of ordinary concrete and LWC was reduced by approximately 35.6% compared to the control sample without bacteria immersed in the water. Therefore, it is also necessary to consider the environmental factor when using microbial self-healing in a degraded environment where urea exists to avoid deterioration. This was also verified by Nodehi et al. [100] who analyzed the precipitation mechanism of bacterial concrete and found that the environmental impact of bacterial concrete was directly related to the urea content in the concrete mixture. Different bacterial types and the environmental impact resulted in different durability performances. They showed that microbial self-healing concrete was even more durable than conventional concrete. These results indicate that the addition of the self-healing admixtures not only improved the self-healing ability of the cracked concrete specimens but also improved the resistance to chloride penetration and possibly prolonged the durability of the non-cracked concrete due to the denser microstructure.

There are many methods for durability testing, including the water permeability test, chloride penetration test, carbonization test, steel corrosion test, etc. However, some tests of durability are not available, such as the durability test of self-healing concrete under freeze-thaw cycles. There are some limitations under these conditions [101]. Although spore bacteria are resistant to lower temperatures (−80 °C), their activity will decrease and their water supply will become slow at low temperatures. Frozen water damages the spores and greatly affects the self-healing process. A non-destructive testing method is wanted in the testing of LWSHC.

## 4. Theoretical Prediction of the Healing Behavior of Lightweight Aggregate Self-Healing Concrete

The theoretical methods such as the finite element method and machine learning method have good ability in prediction and analysis, while the theoretical simulation of LWSHC is still lacking. Zemskov et al. [102] established a mathematical model for the healing of carbonized blast furnace slag cement under accelerated carbonization. They investigated the effect of the impregnation of sodium monofluorophosphate solutions using lightweight expanded clay aggregates [103]. The self-healing process was modeled assuming that the position of the carbonization front varied with time. The model was based on an initial boundary value problem of partial differential equations solved by the Galerkin finite element method. Later, the crack-sealing effect of bio-based healing mortars with expanded clay particles was explored [27]. The research on sealing performance was carried out by experimental and computational methods. Image processing and crack permeability test results were compared with those obtained from computer simulations. Results showed that computer simulations had good prediction results and matched the experimental results, i.e., machine learning methods can provide good predictions for the self-healing of LWSHC. Zhuang and Zhou [104] proposed a model for predicting the crack closure rate of LWSHC based on machine learning and particle swarm optimization. The machine learning algorithm was used to model the nonlinear relationship between the crack closure rate and its influence factors. The performance of the optimal model was verified using the mean squared error and R-value. It was found that the particle swarm optimization algorithm-machine learning method had great potential for the prediction of the crack closure rate of LWSHC. In addition to the influence of water on the entire LWSHC, conditions such as the mix ratio, the stress state of the crack, the stability of the crack state, and the environmental temperature still were needed as input [78,79]. Although there are many experimental results on LWSHC, theoretical models should also be established to reduce material waste and save time when designing new types of LWSHC.

## 5. Conclusions

The research on LWSHC has attracted much attention in recent years. This review has systematically revealed the healing mechanisms, compositions, and performances of LWSHC. The following conclusions are drawn.

Some remarkable achievements have been made in the strength recovery and crack repair of LWSHC. The absorption rate is significantly improved under vacuum conditions. Using a coating technology on LWAs can retain more healing agents in the LWA, which increases the healing effect of LWSHC. Additionally, a low pH matrix can promote the healing effect. Some special treatments, such as high temperatures, on the LWAs are good for improving their properties. Adding healing agents and fibers simultaneously elevates mechanical properties. Using a nutrient solution instead of concrete mixing water is a method of introducing required nutrients when biological healing agents are adopted. Compared to previous research for perlite, vermiculite, zeolite, and silica supports, expanded perlite is the most suitable carrier for bioremediation. Bacillus Licheniformis, Bacillus Megateterium, and Bacillus alcalophilus are superior in inducing calcium carbonate precipitation in LWSHC. The raw materials of the healing agent and the outflow of nutrients lead to an increase in the fluidity of the concrete. The healing agent should therefore be chosen considering the configuration of cracks since the crack-closing ability of different healing agents is different, i.e, as a chemical healing agent, sodium silicate has a higher healing rate than sodium carbonate. Among healing methods, the microbial self-healing method can fill larger cracks, however, the healing is unstable. The biological self-healing system may have more negative aspects than the chemical self-healing system; hence, the chemical healing agents may be better than the biological healing agents in LWSHC at present. Additionally, the non-destructive detection of crack healing is more efficient and convenient for testing LWSHC. The reduction in durability has been partially solved in LWSHC, while the influence of the environment is inescapable. Therefore, theoretical prediction in the field of LWSHC is an emerging research hotspot. The use of LWSHC in construction is a fast and effective method to save materials and reduce costs in material design, thus the future research direction is to promote the application of LWAs in self-healing concrete.

## Figures and Tables

**Table 1:** LWSHC studies with different healing compositions.

Healing Agents	LWA	References
Bacillus pseudofirmus	Expanded clay	[12]
Na_2_CO_3_ solution	Porous ceramsite	[13]
Bacillus subtilis natto	Expanded clay granules	[15]
Paenibacillus mucilaginosus	Expanded vermiculite	[17]
Water	Clinoptilolite zeolite particles	[18]
Na_2_CO_3_ solution	Lightweight clay aggregate	[19]
Sodium silicate solution	Expanded clay	[20]
Sodium silicate solution	Fly ash-based geopolymer foam	[21]
Bacillus sphaericus	Diatomaceous earth, expanded clay, granular activated carbon, metakaolin, zeolite, and air entrainment	[22]
Sporosarcina Halophila	Expanded perlite aggregates	[23]
Bacterium S. pasteurii	Porous and superlight expanded glass	[24]
Alkaliphilic bacteria of the genus Bacillus	Expanded clay granules	[25,26,27]
Sporosarcina pasteurii	Expanded shale aggregate	[28]
Bacillus psuedofirms	Expanded perlite	[29]
Bacillus pseudofirmus	Expanded perlite	[30]
Alkaliphilic bacteria of the genus Bacillus	Expanded clay particles	[31]
Lysinibacillus boronitolerans	Expanded clay	[32]
Sporosarcina pasteurii	Lightweight aggregates of Leca	[33]
Sporosarcina pasteurii	Diatomite	[34]
Sporosarcina Pasteurii, Bacillus Megateterium, Sporosarcina Ureae, and Bacillus Licheniformis	Leca coarse LWA and Leca fine LWA	[35]
Bacillus mucilaginous	Expanded perlite	[36]
Bacillus alkalinitrilicus	Expanded clay particles	[37,38]
Bacillus subtilis	Diatomite pellet	[39]
Bacillus subtilis	Pumice	[40]
Sporosarcina pasteurii	Ceramsite particles	[41,42]
Bacillus alcalophilus	Modified ceramsite particles	[43]
Bacillus mucilaginous	Ceramsite	[44]

**Table 2:** Characteristics of different microorganisms in LWSHC.

Microorganisms	Cell Concentration	References
Alkaliphilic bacteria of the genus Bacillus	10^8^ spores/L	[26]
Sporosarcina pasteurii	10^6^ cells/mL	[33]
Sporosarcina pasteurii	2.36 × 10^8^ cells/mL	[34]
Sporosarcina Pasteurii, Bacillus Megateterium, Sporosarcina Ureae, and Bacillus Licheniformis	10^7^ cells/mL	[35]
Bacillus subtilis	10^9^ cfu/g	[39]
Sporosarcina pasteurii	10^9^ spores/mL	[42]
Bacillus alcalophilus	10^10^–10^11^ cells/mL	[43]
Bacillus mucilaginous	10^8^–10^9^ cells/mL	[44]

**Table 3:** Crack closure performance of different LWAs and different healing agents.

References	Healing Agents	LWA	Crack Closure Rate	Width of Healed Cracks
[12]	Calcium lactate + Bacillus pseudofirmus	Expanded clay	-	220 μm
[13]	Na_2_CO_3_ solution	Porous ceramsite	69.32%	240 μm
[13]	Na_2_CO_3_ solution	Porous ceramsite	60.60%	240 μm
[13]	Na_2_CO_3_ solution	Porous ceramsite	41.74%	240 μm
[13]	Na_2_CO_3_ solution	Porous ceramsite	51.33%	240 μm
[13]	Na_2_CO_3_ solution	Porous ceramsite	64.58%	240 μm
[17]	Paenibacillus mucilaginosus	Expanded vermiculite	63.51%	400 μm
[17]	Paenibacillus mucilaginosus	Expanded vermiculite	98.87%	400 μm
[20]	Sodium silicate	Expanded clay	80%	300 μm
[25]	Alkaliphilic bacteria of the genus Bacillus	Expanded clay granules	86%	300 μm
[34]	Sporosarcina pasteurii	Diatomite	80%	200 μm
[36]	Bacillus mucilaginous	Expanded perlite	98%	850 μm
[37]	Bacillus alkalinitrilicus	Expanded clay particles	61%	460 μm
[42]	Sporosarcina pasteurii	Ceramsite particles	90%	150 μm
[44]	Bacillus mucilaginous	Ceramsite	87.5%	50 μm
[96]	Alkaliphilic bacteria of the genus Bacillus	Expanded clay particles	98%	350 μm

**Table 4:** Summary of the compressive properties of LWSHC.

Healing Agents	LWA	Strength (MPa)	Age	Healing Rate	References
Na_2_CO_3_ solution	Porous ceramsite	24–25.7	3 days	-	[13]
Na_2_CO_3_ solution	Porous ceramsite	29.3–30.9	7 days	-	[13]
Na_2_CO_3_ solution	Porous ceramsite	36.4–38.9	14 days	-	[13]
Na_2_CO_3_ solution	Porous ceramsite	41.2–43.9	28 days	-	[13]
Bacillus subtilis natto	Expanded clay	-	7 days	40%	[15]
Paenibacillus mucilaginosus	expanded vermiculite	20–43	28 days	-	[17]
Water	Clinoptilolite zeolite particles	69.2–80	28 days	-	[18]
Na_2_CO_3_ solution	Lightweight clay aggregate	31.17–31.50	28 days	-	[19]
Sodium silicate	Expanded clay	-	28 days	16–80%	[20]
Diaphorobacter nitroreducens	Diatomaceous earth	59.9 ± 1.4	28 days	-	[22]
Diaphorobacter nitroreducens	Expanded clay	67.8 ± 1.8	28 days	-	[22]
Bacillus sphaericus	Metakaolin	19.6 ± 1.1	28 days	-	[22]
Bacillus sphaericus	Zeolite	49.0 ± 1.4	28 days	-	[22]
Bacterium S. pasteurii	Porous and superlight expanded glass	20–22	7 days	-	[24]
Bacterium S. pasteurii	Porous and superlight expanded glass	25–26	14 days	-	[24]
Bacterium S. pasteurii	Porous and superlight expanded glass	30–32	28 days	-	[24]
Alkaliphilic bacteria of the genus Bacillus	Expanded clay granules	-	28 days	63%	[25]
Alkaliphilic bacteria of the genus Bacillus	Expanded clay aggregates	21–22	28 days	-	[26]
Sporosarcina pasteurii	Lightweight aggregates of Leca	24.70–34.86	28 days	-	[33]
Sporosarcina pasteurii	Lightweight aggregates of Leca	24.80–34.53	14 days	-	[33]
Sporosarcina pasteurii	Lightweight aggregates of Leca	24.29–40.05	90 days	-	[33]
Sporosarcina pasteurii	Lightweight aggregates of Leca	23.91–42.00	180 days	-	[33]
Sporosarcina pasteurii	Lightweight aggregates of Leca	-	28 days	16.5–17.4%	[33]
Sporosarcina pasteurii	Lightweight aggregates of Leca	-	90 days	21.5%	[33]
Sporosarcina pasteurii	Lightweight aggregates of Leca	-	150 days	26.4%	[33]
Sporosarcina Pasteurii	Leca LWA	-	28 days	25.61%	[35]
Bacillus Megateterium	Leca LWA	-	28 days	20.13%	[35]
Sporosarcina Ureae	Leca LWA	-	28 days	16.80%	[35]
Bacillus Licheniformis	Leca LWA	-	28 days	13.98%	[35]
Bacillus subtilis	Diatomite pellet	15	1 day	-	[39]
Bacillus subtilis	Diatomite pellet	26	3 days	-	[39]
Bacillus subtilis	Diatomite pellet	45	28 days	-	[39]
Bacillus subtilis	Diatomite pellet	52	60 days	-	[39]
Bacillus subtilis	Diatomite pellet	57	90 days	-	[39]
Bacillus subtilis	Diatomite pellet	60	365 days	-	[39]
Bacillus subtilis	Diatomite pellet	61	730 days	-	[39]
Bacillus subtilis	Pumice	20–32.7	3 days	-	[40]
Bacillus subtilis	Pumice	24.3–40.7	7 days	-	[40]
Bacillus subtilis	Pumice	30.4–45.4	28 days	-	[40]
Sporosarcina pasteurii	Expanded shale aggregates	43–44	21 days	-	[54]
Sporosarcina pasteurii	Expanded shale aggregates	45–46	49 days	-	[54]
Sporosarcina pasteurii	Expanded shale aggregates	43–44	83 days	-	[54]
Alkaliphilic bacteria of the genus Bacillus	Expanded clay particles	25	28 days	-	[96]
Alkaliphilic bacteria of the genus Bacillus	Expanded clay particles	22	7 d	-	[96]

**Table 5:** Summary of the flexural properties of LWSHC.

Healing Agents	LWA	Strength (MPa)	Age	Healing Rate	References
Na_2_CO_3_ solution	Porous ceramsite	5.07	3 days	-	[13]
Na_2_CO_3_ solution	Porous ceramsite	5.23	3 days	-	[13]
Na_2_CO_3_ solution	Porous ceramsite	5.5	7 days	-	[13]
Na_2_CO_3_ solution	Porous ceramsite	5.73	7 days	-	[13]
Na_2_CO_3_ solution	Porous ceramsite	6.3	14 days	-	[13]
Na_2_CO_3_ solution	Porous ceramsite	6.57	14 days	-	[13]
Na_2_CO_3_ solution	Porous ceramsite	6.97	28 days	-	[13]
Na_2_CO_3_ solution	Porous ceramsite	7.23	28 days	-	[13]
Paenibacillus mucilaginosus	Expanded vermiculite	8.1	28 days	-	[17]
Paenibacillus mucilaginosus	Expanded vermiculite	7.8	28 days	-	[17]
Paenibacillus mucilaginosus	Expanded vermiculite	7.7	28 days	-	[17]
Paenibacillus mucilaginosus	Expanded vermiculite	7.5	28 days	-	[17]
Paenibacillus mucilaginosus	Expanded vermiculite	7.4	28 days	-	[17]
Paenibacillus mucilaginosus	Expanded vermiculite	7.3	28 days	-	[17]
Na_2_CO_3_ solution	Lightweight clay aggregate	6.15	3 days	-	[19]
Na_2_CO_3_ solution	Lightweight clay aggregate	6.04	3 days	-	[19]
Na_2_CO_3_ solution	Lightweight clay aggregate	8.49	28 days	-	[19]
Na_2_CO_3_ solution	Lightweight clay aggregate	8.62	28 days	-	[19]
Sodium silicate solution	Expanded clay	-	28days	80%	[20]
Bacillus subtilis	Diatomite pellet	4	1 day	-	[39]
Bacillus subtilis	Diatomite pellet	5	3 days	-	[39]
Bacillus subtilis	Diatomite pellet	5	7 days	-	[39]
Bacillus subtilis	Diatomite pellet	6	14 days	-	[39]
Bacillus subtilis	Diatomite pellet	7	28 days	-	[39]
Bacillus subtilis	Diatomite pellet	8	60 days	-	[39]
Bacillus subtilis	Diatomite pellet	8	90 days	-	[39]
Bacillus subtilis	Diatomite pellet	9	365 days	-	[39]
Bacillus subtilis	Diatomite pellet	9	730 days	-	[39]
Bacillus subtilis	Pumice	6.91	28 days	-	[40]
Bacillus subtilis	Pumice	7.73	28 days	-	[40]
Bacillus subtilis	Pumice	8.67	28 days	-	[40]
Bacillus subtilis	Pumice	7.27	28 days	-	[40]
Bacillus subtilis	Pumice	7.5	28 days	-	[40]
Bacillus subtilis	Pumice	7.27	28 days	-	[40]
Bacillus subtilis	Pumice	8.32	28 days	-	[40]
Bacillus subtilis	Pumice	8.91	28 days	-	[40]
Bacillus subtilis	Pumice	8.09	28 days	-	[40]
Bacillus subtilis	Pumice	8.55	28 days	-	[40]
Sporosarcina pasteurii	Ceramsite particles	-	120 days	10%	[42]
Sporosarcina pasteurii	Ceramsite particles	-	120 days	5%	[42]
Sporosarcina pasteurii	Ceramsite particles	-	120 days	17%	[42]
Bacillus mucilaginous	Ceramsite	-	28 days	56%	[44]
Bacillus mucilaginous	Ceramsite	-	28 days	72%	[44]
Alkaliphilic bacteria of the genus Bacillus	Expanded clay particles	5.5	28 days	-	[96]
Alkaliphilic bacteria of the genus Bacillus	Expanded clay particles	5.7	7 days	-	[96]

**Table 6:** Summary of the tensile properties of LWSHC.

Healing Agents	LWA	Strength (MPa)	Age	Healing Rate	References
Water	Clinoptilolite zeolite particles	7.54	28 days	-	[18]
Water	Clinoptilolite zeolite particles	7.56	28 days	-	[18]
Water	Clinoptilolite zeolite particles	7.8	28 days	-	[18]
Water	Clinoptilolite zeolite particles	7.7	28 days	-	[18]
Water	Clinoptilolite zeolite particles	7.98	28 days	-	[18]
Water	Clinoptilolite zeolite particles	-	28 days	98%	[18]
Bacterium S. pasteurii	Porous and superlight expanded glass	3	7 days	-	[24]
Bacterium S. pasteurii	Porous and superlight expanded glass	3.1	7 days	-	[24]
Bacterium S. pasteurii	Porous and superlight expanded glass	3.8	14 days	-	[24]
Bacterium S. pasteurii	Porous and superlight expanded glass	3.9	14 days	-	[24]
Bacterium S. pasteurii	Porous and superlight expanded glass	4.1	28 days	-	[24]
Bacterium S. pasteurii	Porous and superlight expanded glass	4.2	28 days	-	[24]

## Data Availability

Data are contained within the article.
